# Evaluation of the *XRCC1* gene as a phenotypic modifier in *BRCA1/2* mutation carriers. Results from the consortium of investigators of modifiers of *BRCA1/BRCA2*

**DOI:** 10.1038/bjc.2011.91

**Published:** 2011-03-22

**Authors:** A Osorio, R L Milne, R Alonso, G Pita, P Peterlongo, A Teulé, K L Nathanson, S M Domchek, T Rebbeck, A Lasa, I Konstantopoulou, F B Hogervorst, S Verhoef, M F van Dooren, A Jager, M G E M Ausems, C M Aalfs, C J van Asperen, M Vreeswijk, Q Waisfisz, C E Van Roozendaal, M J Ligtenberg, HEBON, EMBRACE, D F Easton, S Peock, M Cook, C T Oliver, D Frost, B Curzon, D G Evans, F Lalloo, R Eeles, L Izatt, R Davidson, J Adlard, D Eccles, K-r Ong, F Douglas, S Downing, C Brewer, L Walker, H Nevanlinna, K Aittomäki, F J Couch, Z Fredericksen, N M Lindor, A Godwin, C Isaacs, M A Caligo, N Loman, H Jernström, G Barbany-Bustinza, A Liljegren, H Ehrencrona, M Stenmark-Askmalm, Sw E-BRCA, L Feliubadaló, S Manoukian, B Peissel, D Zaffaroni, B Bonanni, S Fortuzzi, O T Johannsson, G Chenevix-Trench, X-C Chen, J Beesley, A B Spurdle, kConFab, O M Sinilnikova, S Healey, L McGuffog, A C Antoniou, J Brunet, P Radice, J Benítez, on behalf of CIMBA

**Affiliations:** 1 Human Genetics Group, Spanish National Cancer Centre, C/Melchor Fernández Almagro 3, 28029 Madrid, Spain; 2 Spanish Network on Rare Diseases (CIBERER); 3 Genetic and Molecular Epidemiology Group, Spanish National Cancer Centre, Madrid, Spain; 4 Genotyping Unit, Human Cancer Genetics Programme, Spanish National Cancer Centre, Madrid, Spain; 5 Unit of Molecular bases of genetic risk and genetic testing, Department of Preventive and Predictive Medicine, Fondazione IRCCS Istituto Nazionale Tumori, Milan, Italy; 6 IFOM, Fondazione Istituto FIRC di Oncologia Molecolare, Milan, Italy; 7 Hereditary Cancer Program, Catalan Institute of Oncology, Spain; 8 Abramson Cancer Center, University of Pennsylvania, Philadelphia, PA, USA; 9 Genetics Service, Hospital de la Santa Creu i Sant Pau, Barcelona, España; 10 Molecular Diagnostics Lab IRRP, NCSR ‘Demokritos’, 15310 Agia Paraskevi, Greece; 11 Family Cancer Clinic, Netherlands Cancer Institute, Amsterdam, The Netherlands; 12 Department of Clinical Genetics, Netherlands Cancer Institute, Amsterdam, The Netherlands; 13 Department of Clinical Genetics, Family Cancer Clinic, Erasmus University Medical Center, Rotterdam, The Netherlands; 14 Department of Medical Oncology, Family Cancer Clinic, Erasmus University Medical Center, Rotterdam, The Netherlands; 15 Department of Medical Genetics, University Medical Center Utrecht, Utrecht, The Netherlands; 16 Department of Clinical Genetics, Academic Medical Center, Amsterdam, The Netherlands; 17 Department Of Clinical Genetics, Leiden University Medical Center, Leiden, The Netherlands; 18 Department of Human Genetics and Department of Toxicogenetics, Leiden University Medical Center, Leiden, The Netherlands; 19 Department of Clinical Genetics, VU University Medical Center, Amsterdam, The Netherlands; 20 Department of Clinical Genetics, University Medical Center, Maastricht, The Netherlands; 21 Department of Human Genetics and Pathology, Radboud University Nijmegen Medical Centre, Nijmegen, The Netherlands; 22 The Hereditary Breast and Ovarian Cancer Research Group Netherlands; 23 Centre for Cancer Genetic Epidemiology, Department of Public Health and Primary Care, University of Cambridge, UK; 24 Centre for Cancer Genetic Epidemiology, Department of Oncology, University of Cambridge, UK; 25 Genetic Medicine, Manchester Academic Health Sciences Centre, Central Manchester University Hospitals NHS Foundation Trust, Manchester, UK; 26 Oncogenetics Team, The Institute of Cancer Research and Royal Marsden NHS Foundation Trust, UK; 27 Clinical Genetics, Guy's and St. Thomas’ NHS Foundation Trust, London, UK; 28 Ferguson-Smith Centre for Clinical Genetics, Yorkhill Hospitals, Glasgow, UK; 29 Yorkshire Regional Genetics Service, Leeds, UK; 30 Wessex Clinical Genetics Service, Princess Anne Hospital, Southampton, UK; 31 West Midlands Regional Genetics Service, Birmingham Women's Hospital Healthcare NHS Trust, Edgbaston, Birmingham, UK; 32 Institute of Human Genetics, Centre for Life, Newcastle Upon Tyne Hospitals NHS Trust, Newcastle upon Tyne, UK; 33 Department of Clinical Genetics, East Anglian Regional Genetics Service, Addenbrookes Hospital, Cambridge, UK; 34 Department of Clinical Genetics, Royal Devon and Exeter Hospital, Exeter, UK; 35 Oxford Regional Genetics Service, Churchill Hospital, Oxford, UK; 36 Department of Obstetrics and Gynecology, Helsinki University Central Hospital, Helsinki, Finland; 37 Clinical Genetics, Helsinki University Central Hospital, Helsinki, Finland; 38 Department of Laboratory Medicine and Pathology, Mayo Clinic, Rochester, MI, USA; 39 Department of Health Sciences Research, Mayo Clinic, Rochester, MI, USA; 40 Department of Medical Genetics, Mayo Clinic, Rochester, MI, USA; 41 Department of Pathology and Laboratory Medicine, University of Kansas Medical Center, KS, USA; 42 MD Georgetown University, Washington, DC, USA; 43 Division of Pathology, Department of Oncology, University and University Hospital of Pisa, Italy; 44 Department of Oncology, Lund University Hospital, Lund, Sweden; 45 Department of Oncology, Lund University Hospital, Lund, Sweden; 46 Department of Clinical Genetics, Karolinska University Hospital, S-17176 Stockholm, Sweden; 47 Department of Oncology, Karolinska University Hospital, Stockholm, Sweden; 48 Department of Genetics and Pathology, Rudbeck Laboratory, Uppsala University, S-751 85 Uppsala, Sweden; 49 Department of Oncology, University Hospital Linköping, Sweden; 50 Unit of Medical Genetics, Department of Preventive and Predictive Medicine, Fondazione IRCCS Istituto Nazionale dei Tumori, Milan, Italy; 51 Division of Cancer Prevention and Genetics, Istituto Europeo di Oncologia, Milan, Italy; 52 Cogentech, Consortium for Genomics Technology, Milan, Italy; 53 Faculty of Medicine, University of Iceland, Department of Oncology, Landspitali-University Hospital, Reykjavik, Iceland; 54 Genetics and Population Health Division, Queensland Institute of Medical Research, Brisbane, Australia; 55 Peter MacCallum Cancer Centre, Melbourne, Australia; 56 Unité Mixte de Génétique Constitutionnelle des Cancers Fréquents, Centre Hospitalier Universitaire de Lyon/Centre Léon Bérard, Lyon, France; 57 Equipe labellisée LIGUE 2008, UMR5201 CNRS, Centre Léon Bérard, Université de Lyon, Lyon, France

**Keywords:** *BRCA1*, *BRCA2*, *XRCC1*, breast cancer

## Abstract

**Background:**

Single-nucleotide polymorphisms (SNPs) in genes involved in DNA repair are good candidates to be tested as phenotypic modifiers for carriers of mutations in the high-risk susceptibility genes *BRCA1* and *BRCA2.* The base excision repair (BER) pathway could be particularly interesting given the relation of synthetic lethality that exists between one of the components of the pathway, *PARP1*, and both *BRCA1* and *BRCA2*. In this study, we have evaluated the *XRCC1* gene that participates in the BER pathway, as phenotypic modifier of *BRCA1* and *BRCA2*.

**Methods:**

Three common SNPs in the gene, c.-77C>T (rs3213245) p.Arg280His (rs25489) and p.Gln399Arg (rs25487) were analysed in a series of 701 *BRCA1* and 576 *BRCA2* mutation carriers.

**Results:**

An association was observed between p.Arg280His-rs25489 and breast cancer risk for *BRCA2* mutation carriers, with rare homozygotes at increased risk relative to common homozygotes (hazard ratio: 22.3, 95% confidence interval: 14.3–34, *P*<0.001). This association was further tested in a second series of 4480 *BRCA1* and 3016 *BRCA2* mutation carriers from the Consortium of Investigators of Modifiers of *BRCA1* and *BRCA2*.

**Conclusions and inte:**

No evidence of association was found when the larger series was analysed which lead us to conclude that none of the three SNPs are significant modifiers of breast cancer risk for mutation carriers.

Germ-line mutations in the *BRCA1* and *BRCA2* genes confer a high lifetime risk of developing breast or ovarian cancer. Estimates of the cumulative risk of breast cancer to age 70 vary from 40 to 85%, depending on the study (Easton *et al*, 1995; Ford *et al*, 1998; Antoniou *et al*, 2003; Chen *et al*, 2006; Milne *et al*, 2008). Environmental and other genetic factors (risk modifiers) are likely to explain these differences, at least in part. The few reliable genetic associations that have been reported to date, have all come from the Consortium of Investigators of Modifiers of *BRCA1/2* (CIMBA) initiative, which was set up to provide large samples of mutation carriers to reliably assess even modest associations with single-nucleotide polymorphisms (Chenevix-Trench *et al*, 2007). The CIMBA has assessed risk in *BRCA1/2* carriers for various SNPs in genes that had been previously found to be associated with increased breast cancer risk in the general population, mostly via genome-wide association studies (Antoniou *et al*, 2008, 2009). However, the first evidence of a modifier came from a candidate gene approach studying the *RAD51* gene, which interacts directly with *BRCA1* and *BRCA2*. All three genes participate in the DNA double-strand break repair by the homologous recombination pathway. Results from the CIMBA study suggested an increased risk of breast cancer for *BRCA2* mutation carriers with two copies of the ‘C’ allele at the 135G → C SNP (rs 1801320) in the 5′ untranslated region of *RAD51* (Antoniou *et al*, 2007). This result suggests that other genes involved in DNA repair could as also function as cancer risk modifiers for *BRCA1* and *BRCA2* mutation carriers.

It has been proven that a deficiency in the base excision repair (BER) pathway can give rise to stalling of the replication fork and accumulation of double-strand DNA breaks which, in the presence of a defective *BRCA1* or *BRCA2* background, could persist and lead to cell cycle arrest or cell death (Farmer *et al*, 2005). This synthetic lethality interaction led us to hypothesise that SNPs in genes participating in this pathway could be potential modifiers of cancer risk in *BRCA1* and *BRCA2* mutation carriers. The *XRCC1* gene is involved in the BER pathway and its association with different types of cancer have been extensively investigated, with no conclusive results (Kiyohara *et al*, 2006; Figueroa *et al*, 2007; Naccarati *et al*, 2007; Doecke *et al*, 2008; Fontana *et al*, 2008; McWilliams *et al*, 2008; Chang *et al*, 2009; Zhai *et al*, 2009). Four SNPs in *XRCC1*, three of them leading to amino acid changes (p.Arg194Trp, p.Arg280His and p.Gln399Arg) and one in the promoter region (c.-77C>T) are within the most common in terms of minor allele frequency, and a potential effect on the function of the XRCC1 protein has been suggested for them, although no clear association with breast cancer risk has been reported (Takanami *et al*, 2005; Hao *et al*, 2006; Sterpone *et al*, 2009; Sterpone *et al*, 2010). Nevertheless, the specific interaction mentioned above suggests that common variation in *XRCC1* may have an effect on breast cancer risk for *BRCA1* or *BRCA2* mutation carriers. In this study, we aimed to assess this hypothesis for the three most studied SNPs in *XRCC1*, c.-77C>T (rs3213245) p.Arg280His (rs25489) and p.Gln399Arg (rs25487) using a two-stage approach.

## Material and methods

### Patients

Eligible subjects were female carriers of deleterious mutations in *BRCA1* or *BRCA2* aged ≥18 years from which complete information about year of birth, mutation description, age at last follow-up, ages at breast and or ovarian cancer diagnosis and age or date of prophylactic mastectomy was available (Antoniou *et al*, 2008). A total of 14 collaborating CIMBA studies from 10 countries, contributed genotypes for the study. Details of each study along with the numbers of samples included from each are provided in Table 1. The CNIO, ICO and MBCSG studies participated in the first stage in which the three SNPs, rs3213245, rs25489 and rs25487 were analysed and a potential association between rs25489 and breast cancer risk was identified. The remaining CIMBA samples were included in the stage II analysis and contributed genotypes for rs25489 only.

Subjects who reported having ethnicity other than white European were excluded from the analyses. This gave a total of 7496 female mutation carriers (4480 with mutations in *BRCA1* and 3016 with mutations in *BRCA2*), 3891 of whom had been diagnosed with breast cancer (2293 and 1598 with mutations in *BRCA1* and *BRCA2*, respectively). All carriers participated in clinical and/or research studies at the host institution under IRB-approved protocols.

### Genotyping

The genotyping platform used by each study is detailed in Table 1. For 11 studies, matrix assisted laser desorption/ionisation time of flight mass spectrometry was applied to determine allele-specific primer extension products using Sequenom's MassARRAY system and iPLEX technology (Sequenom, San Diego, CA, USA). The design of oligonucleotides was carried out according to the guidelines of Sequenom and performed using MassARRAY Assay Design software (version 3.1). Three studies carried out genotyping by nuclease assay (Taqman). Taqman genotyping reagents were designed by Applied Biosystems (http://www.appliedbiosystems. com/) as Assays-by-Design. Genotyping was performed using the ABI PRISM 7900HT, 7700 or 7500 Sequence Detection Systems according to manufacturer's instructions. All studies complied with CIMBA genotyping quality control standards (http://www.srl.cam.ac.uk/consortia/cimba/eligibility/eligibi lity.html).

### Statistical analysis

To test for departure from Hardy–Weinberg equilibrium a single individual was randomly selected from each family and Pearson's *χ*^2^-test (1 d.f.) was applied to genotypes from this set of individuals. The association of the SNPs with breast cancer risk was assessed by estimating hazard ratios (HRs) and their corresponding 95% confidence intervals (CIs) using weighted multivariable Cox proportional hazards regression with robust estimates of variance (Antoniou *et al*, 2005). For each mutation carrier, we modelled the time to diagnosis of breast cancer from birth, censoring at the first of the following events: bilateral prophylactic mastectomy, breast cancer diagnosis, ovarian cancer diagnosis, death and date last know to be alive. Subjects were considered affected if their age at censoring corresponded to their age at diagnosis of breast cancer and unaffected otherwise. Weights were assigned separately for carriers of mutations in *BRCA1* and *BRCA2*, by age (<25, 25–29, 30–34, 35–39, 40–44, 45–49, 50–54, 55–59, 60–64, 65–69, ⩾70) and affection status, so that the weighted observed incidences in the sample agreed with established estimates for mutation carriers (Antoniou *et al*, 2003). This approach has been shown to adjust for the bias inherent in the oversampling of affected women because of the ascertainment criteria used (Antoniou *et al*, 2005).

We considered log-additive and co-dominant genetic models and tested for departure from HR=1 by applying a Wald test based on the log−HR estimate and its standard error. Additional independent variables included in all analyses were year of birth (<1930, 1930–1939, 1940–1949, 1950–1959, 1960–1969, ⩾1970), study centre and country. Heterogeneity in HRs by study centre was assessed by the *χ*^2^ equivalent of a Wald test based on the interaction terms for the per-allele effect by centre (on 13 d.f.). A number of sensitivity analyses were applied, including censoring at bilateral prophylactic oophorectomy (BPO), adjusting for BPO (as a time-varying covariate) and excluding prevalent cases, defined as those diagnosed more than 3 years before the interview.

All statistical analyses were carried out using Stata: Release 10 (StataCorp. 2007. Stata Statistical Software: Release 10.0. College Station, TX, USA: Stata Corporation LP). Robust estimates of variance were calculated using the cluster sub-command, applied to an identifier variable unique to each family.

## Results and discussion

In this study, we aimed to evaluate the role of three of the most studied SNPs in the *XRCC1* gene, c.-77C>T (rs3213245) p.Arg280His (rs25489) and p.Gln399Arg (rs25487) as modifiers of breast cancer risk in *BRCA1* and *BRCA2* mutation carriers. The study was conducted in two stages, the first analysing the three SNPs in 1277 mutation carries (701 in *BRCA1* and 576 in *BRCA2*) from three CIMBA study centres (CNIO, ICO and MBCSG). No evidence of association was detected for c.-77C>T or p.Gln399Arg with breast cancer risk in neither *BRCA1* nor *BRCA2* mutation carriers (*P*⩾0.2). However, an association was observed between p.Arg280His and breast cancer risk for *BRCA2* mutation carriers, with rare homozygotes at increased risk relative to common homozygotes (HR: 22.3, 95% CI: 14.3–34, *P*<0.001; Table 2). The apparent increased risk was consistent with the fact that the 280His allele decreases DNA repair capacity (Takanami *et al*, 2005; Pachkowski *et al*, 2006), however the analysis was based on a very small number (*N*=2) of homozygous women diagnosed at a very early age and it was therefore essential that this result be investigated in a larger sample set.

We therefore extended the analysis of the p.Arg280His-rs25489 SNP to 6219 carriers from 11 additional CIMBA study centres. Results from stage II and both stages combined are summarised in Table 2. No evidence of an association of AA *vs* GG homozygotes with breast cancer risk was observed for *BRCA2* mutation carriers (HR: 0.73, 95% CI: 0.21–2.52, *P*=0.6 in stage II and HR: 1.08, 95% CI: 0.37–3.17, *P*=0.9 in the combined), nor for *BRCA1* mutation carriers or all mutation carriers combined. We observed no evidence of between-study heterogeneity for carriers of mutations in *BRCA2* (*P*=0.8). There was evidence of heterogeneity in the per-allele HR for *BRCA1* mutation carriers (*P*=0.006); exclusion of subjects from potential outlier studies did not eliminated this evidence (*P*<0.05) and the estimated HR estimate did not change substantially. Several sensitivity analyses were carried out (see Materials and methods), but results did not change substantially and so only those from the main analysis are presented in this report.

Our results do not provide support for the hypothesis that the three most common and putatively functional SNPs in *XRCC1* modify breast cancer risk for *BRCA1* and *BRCA2* mutation carriers. However, given the demonstrated interaction that exists between the homologous recombination and BER DNA repair pathways, additional SNPs in *XRCC1* and other genes involved in BER should be assessed as risk modifiers for *BRCA1/2* mutation carriers in future studies.

## Figures and Tables

**Table 1:** Number of *BRCA1* and *BRCA2* mutation carriers by study

**Study**	**Country of residence**	***BRCA1***	***BRCA2***	**Genotyping platform**
Spanish National Cancer Centre^a^^,^^b^	Spain and Greece^a^	144	147	Taqman
Catalan Institute of Oncology^b^	Spain	144	177	Taqman
Hereditary Breast and Ovarian study Netherlands	The Netherlands	791	308	iPlex
Epidemiological study of BRCA1 and BRCA2 mutation carriers	UK and Eire	997	817	iPlex
Fox Chase Cancer Center	USA	83	54	iPlex
Georgetown	USA	43	35	iPlex
Helsinki Breast Cancer Study	Finland	103	104	iPlex
Iceland Landspitali – University Hospital	Iceland	0	133	iPlex
Kathleen Cuningham Consortium for Research into Familial Breast Cancer	Australia	592	478	iPlex
Mayo Clinic	USA	231	126	iPlex
Milan Breast Cancer Study Group^b^	Italy	413	252	Taqman
Pisa Breast Cancer Study	Italy	86	56	iPlex
Swedish Breast Cancer Study	Sweden	536	176	iPlex
University of Pennsylvania	USA	317	153	iPlex
Total^c^		4480	3016	

a The Spanish National Cancer Centre series consisted of mutation carriers from the Spanish Consortium for the Study of Genetic Modifiers of *BRCA1* and *BRCA2* and the National Centre for Sensor Research Demokritos, Athens and Greece.

b Series included in stage I of the study.

c Mutation carriers that failed genotyping are not included in the totals.

**Table 2:** Genotype frequencies of *XRCC1*-rs25489 by mutation and disease status and hazard ratio estimates from stages I and II and combined

	**Genotype**	**Unaffected (%)**	**Affected (%)**	**HR**	**95% CI**	***P*-value**
*Stage I*						
BRCA1 (*n*=701)	GG	300 (89.8)	317 (86.4)	1.00		
	AG	33 (9.88)	49 (13.4)	1.29	0.85–1.97	0.2
	AA	1 (0.30)	1 (0.27)	0.87	0.24–3.20	0.8
BRCA2 (*n*=576)	GG	226 (88.3)	283 (88.4)	1.00		
	AG	30 (11.7)	35 (10.9)	1.20	0.69–2.08	0.5
	**AA**	**0**	**2 (0.63)**	**22.3**	**14.6–34.0**	**<0.001**
						
*Stage II*						
BRCA1 (*n*=4480)	GG	1659 (89.5)	1757 (91.2)	1.00		
	AG	192 (10.4)	166 (8.62)	0.83	0.67–1.02	0.07
	AA	2 (0.11)	3 (0.16)	1.26	0.30–5.32	0.8
BRCA2 (*n*=3016)	GG	1045 (89.9)	1143 (89.2)	1.00		
	AG	112 (9.64)	131 (10.3)	0.98	0.77–1.25	0.9
	AA	5 (0.43)	4 (0.31)	0.73	0.21–2.52	0.6
						
*Combined*						
BRCA1 (*n*=5181)	GG	1959 (89.6)	2074 (90.5)	1.00		
	AG	225 (10.3)	215 (9.38)	0.89	0.74–1.07	0.2
	AA	3 (0.14)	4 (0.17)	0.72	0.20–2.60	0.6
BRCA2 (*n*=3592)	GG	1271 (89.6)	1426 (89.2)	1.00		
	AG	142 (10)	166 (10.4)	1.02	0.81–1.28	0.9
	AA	5 (0.35)	6 (0.38)	1.08	0.37–3.14	0.9

Abbreviations: CI=confidence interval; HR=hazard ratio. Statistically significant results are highlighted in bold.
